# Subaxial lordosis loss and influence factors after posterior atlantoaxial fusion

**DOI:** 10.1186/s13018-022-03077-6

**Published:** 2022-03-28

**Authors:** Shaoqiang Liu, Boling Liu, Guiqing Liang, Qiyong Chen, Huafeng Wang, Yuhan Lin

**Affiliations:** Department of Spine Surgery, Fuzhou Second Hospital of Xiamen University, School of Medicine, Xiamen University (Fuzhou Second Hospital), No. 47 Shangteng Rd, Cangshan District, Fuzhou, 350007 China

**Keywords:** Atlantoaxial fusion, Subaxial lordosis, Cervical sagittal balance, Factor analysis

## Abstract

**Summary of background data:**

Cervical sagittal balance is an important evaluation index of cervical physiological function and surgical efficacy. Subaxial kyphosis after atlantoaxial fusion is negatively associated with worse clinical outcomes and higher incidence of lower cervical disk degeneration.

**Objectives:**

This study aimed to confirm the factors that influence subaxial lordosis loss after posterior atlantoaxial fusion.

**Methods:**

We performed a retrospective review of all patients following posterior C1–C2 fusion for atlantoaxial dislocation between January 2015 and December 2017. All charts, records, and imaging studies were reviewed for each case, and preoperative, immediate postoperative, and final follow-up plain films were evaluated. Comparing final follow-up and preoperative C2–C7 angle, patients were divided into two groups for further comparison: subaxial lordosis loss group and subaxial lordosis increase group.

**Results:**

A total of 18 patients were included in the review, with an average radiographic follow-up of 8.4 ± 3.7 months (range 6–17 months). Subaxial lordosis loss was observed in 5 cases (27.8%) at the final follow-up, whereas 13 cases had an increase in subaxial lordosis. The cervical sagittal parameters of preoperative and final follow-up between two groups were compared, the preoperative C2–C7 angle of the subaxial lordosis loss group was bigger than the subaxial lordosis increase group (27.6° ± 10.5° vs 10.5° ± 10.5°, *P* < 0.05), but there was no statistical difference in other parameters. Univariate chi-square analysis showed that reduction in subaxial lordosis after posterior atlantoaxial fusion was associated with preoperative C2–C7 angle ≥ 20° (*χ*^2^ = 4.923, *P* = 0.026). However, Logistic regression analysis showed that the preoperative C2–C7 angle ≥ 20° was not an independent risk factor (OR = 0.147, *P* = 0.225).

**Conclusion:**

Our study demonstrates that subaxial lordosis loss may occur after posterior atlantoaxial fusion, and preoperative C2–C7 angle ≥ 20° was a risk factor of postoperative loss of subaxial lordosis.

## Introduction

Atlantoaxial dislocation (AAD) is a rare disorder of the craniocervical junction characterized by C1–C2 instability and loss of normal articulation. It is often associated with complex deformities of the craniovertebral junction and poses a significant risk of neurological deterioration [1]. The unique position of the odontoid rising between the ring of the atlas ventrally, the transverse atlantal ligament dorsally and the C1–C2 lateral mass joints are the major factors in preventing dislocation of C1 and C2. Any disruption of the integrity of the odontoid or the transverse atlantal ligament would predispose to AAD. With regards to its etiology, AAD can be divided into three main categories: traumatic, idiopathic, and deformity-related [2, 3].

Posterior atlantoaxial fusion is the main surgical method for the treatment of atlantoaxial dislocation. It has achieved good clinical results and fusion rate [4]. However, some patients after atlantoaxial fusion may develop loss of normal lordotic curvature of the cervical spine, straight cervical alignment, kyphotic deformity, and even swan neck deformity, accompanying with anterior moving of the inferior articular process, tear of the joint capsule, increased height between spinous processes, resulting in subluxation [4–6]. Loss of subaxial lordosis often refers to reduction in lower cervical lordosis (C2–C7 angle) or increase in kyphosis after surgery [7–9], while increased lordosis means that the postoperative C2–C7 angle is larger than preoperative C2–C7 angle.

Cervical sagittal balance, an important evaluation index of cervical physiological function and surgical efficacy, has received increasing attention recently [10]. Studies found that cervical sagittal alignment is related to postoperative outcomes for patients receiving multilevel cervical posterior fusion, and the severity of disability increases with positive sagittal malalignment following surgical reconstruction [11]. Loss of subaxial lordosis after posterior atlantoaxial fusion also has some influence on clinical outcomes. Subaxial kyphosis after atlantoaxial fusion is negatively associated with worse clinical outcomes and higher incidence of lower cervical disk degeneration [12]. Subaxial kyphosis or segmental instability after atlantoaxial fusion in patients with rheumatoid arthritis may result in secondary myelopathy, requiring reoperation [13, 14]. In this study, we measured the changes of cervical sagittal parameters and evaluated the related factors after posterior atlantoaxial fusion.

## Methods

### Patient recruitment

Following institutional review board (IRB) approval, we evaluated the retrospectively collected single-institute database of patients who had posterior atlantoaxial fusion for atlantoaxial dislocation between January 2015 and December 2017. Among those patients, we applied the following inclusion criteria to identify the study cohort: (1) patients diagnosed with atlantoaxial dislocation, (2) patients with Wang classification [15] Type I (instability) and Type II (reducible dislocation), (3) patients who had a minimum radiographic follow-up of 6 months, and (4) patients who were treated with the same standard posterior reduction and C1–C2 fixation. In addition, the following exclusion criteria were applied: (1) patients with posterior or vertical atlantoaxial dislocation, (2) patients with occipitocervical junction deformity, such as basilar invagination, (3) patients with cervical tuberculosis, infection or tumor, (4) patients with ankylosing spondylitis, (5) patients with prior cervical spine surgery, and (6) patients with severe medical comorbidity.

Applying these criteria yielded a total of 18 patients that were included in our final analysis. There were 8 males and 10 females with an average age of 49.6 ± 13.7 years (range 25–72 years). Among the patients, 15 had transverse ligament loosen, 2 os odontoideum, and 1 rheumatoid polyarthritis. The study is a retrospective radiographic analysis.

### Surgical techniques

Types I and II atlantoaxial dislocation were relatively easily fixed and fused in the reduced position from a posterior approach. Through a midline incision, we exposed the posterior arch of the atlas and the lamina of C2. The vessels and nerve roots of C2 were pulled caudally. The junction between the lateral mass and the inferior arch of C1 was exposed. Then we visualized and palpated the pedicle of C1. A point was opened with a burr. The pedicle of C1 was prepared with a 2.5-mm drill bit. After tapping a 3.5 mm pedicle screw (usually 28–30 mm in length) was inserted into each pedicle. Next, the vessels and nerve roots of C2 were retracted cephalad to expose the superior and internal aspect of the isthmus of C2. The C2 pedicle screw (diameter, 3.5 mm; length, 22–26 mm) was drilled, tapped, and inserted using the technique described by Abumi et al. [14]. Two rods were placed, and morselized cancellous graft harvest from the posterior superior iliac spine was used.

### Radiologic evaluation

Radiologic evaluation included standard lateral view of radiographs in a neutral position, which was taken preoperatively, immediate postoperatively and at final follow-up. For every patient, two senior authors performed the measurements twice independently. Measurements were performed using Cobb’s technique [16].

**Fig. 1:**
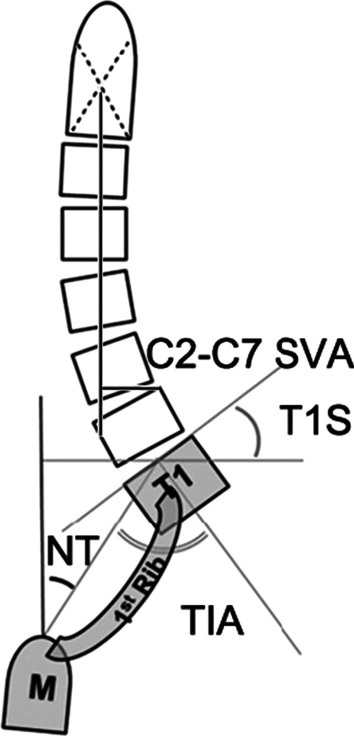
Cervical sagittal parameters (C2–C7 SVA, C2–C7 sagittal vertical axis; T1S, T1 slope; NT, neck tilt; TIA, thoracic inlet angle; T1, first thoracic vertebra; M, manubrium; 1st Rib, first rib)

C0–C2 lordosis was measured as the angle between the McGregor line and lower endplate of C2. The measurement of C1–C2 lordosis was calculated between a line connecting the anterior tubercle to the posterior margin of the C1 spinous process and the inferior end plate of C2. C2–C7 lordosis was defined as the Cobb angle between the lower endplates of C2 and C7. C0–C7 angle was measured as the angle between the McGregor line and lower endplate of C7. The C2–C7 sagittal vertical axis (SVA) was measured as the distance from the posterosuperior corner of C7 and the vertical line from the center of the C2 body. The T1 slope (T1S) was the angle formed by the tangent to the upper endplate of T1 and the horizontal reference line. The neck tilt (NT) was defined as an angle formed by a line drawn in the upper end of the sternum and a line connecting the center of the upper endplate of T1 and the upper end of the sternum. The thoracic inlet angle (TIA) as an angle formed by a line from the center of the T1 upper end plate vertical to the T1 upper end plate and a line connecting the center of the T1 upper end plate and the upper end of the sternum (Fig. 1).

### Grouping

At the last follow-up, patients were divided into two groups for further comparison: subaxial lordosis loss group (*n* = 5) and subaxial lordosis increase group (*n* = 13). The subaxial lordosis loss group included the patients whose final follow-up C2–C7 angle was reduced compared with preoperative C2–C7 angle, while the subaxial lordosis increase group included the patients whose final follow-up C2–C7 angle increased.

### Statistical analysis

The t test was used to compare the pre-operative and final follow-up cervical sagittal parameters, and compare the cervical sagittal parameters between two groups before operation and final follow-up. Binary logistic regression was used for continuous data, and Cochran and Mantel–Haenszel chi-squared tests were used for dichotomous data. Univariate and multivariate logistic regression analyses were used to identify potential risk factors. Multivariate logistic regression was performed using variables with *P* < 0.20 in the univariate analysis. Statistical significance was considered as *P* < 0.05. All the statistical analyses were performed using SPSS ver. 16.0 statistical software (SPSS, Chicago, IL, USA).

## Results

A total of 18 patients were included in the review, with an average radiographic follow-up of 8.4 ± 3.7 months (range 6–17 months). Overall, subaxial lordosis loss was observed in 5 cases (27.8%) at the final follow-up, whereas 13 cases had an increase in subaxial lordosis. The comparison of the cervical sagittal parameters between pre-operation and final follow-up is shown in Table 1. There were no significant changes in C0–C2 angle, C2–C7 angle, C0–C7 angle, C1–C2 angle, C2–C7 SVA, T1S, NT, and TIA at the last follow-up and pre-operative (*P* > 0.05).

**Table 1:** Comparison of the cervical sagittal parameters between pre-operation and final follow-up

	Pre-operation	Final follow-up	*t* value	*P* value
C0–C2 (°)	21.6 ± 16.4	28.3 ± 8.6	− 1.536	0.137
C1–C2 (°)	12.4 ± 17.6	17.5 ± 7.3	− 1.124	0.273
C2–C7 (°)	15.3 ± 12.9	16.4 ± 11.1	− 0.292	0.772
C0–C7 (°)	36.8 ± 19.7	44.9 ± 13.2	− 1.453	0.157
C2–C7 SVA (mm)	13.4 ± 14.7	15.1 ± 11.7	− 0.376	0.709
T1S (°)	22.8 ± 8.2	23.5 ± 7.3	− 0.258	0.798
NT (°)	50.8 ± 9.5	51.9 ± 8.9	− 0.361	0.720
TIA (°)	73.6 ± 11.1	75.4 ± 10.0	− 0.505	0.617

**Fig. 2:**
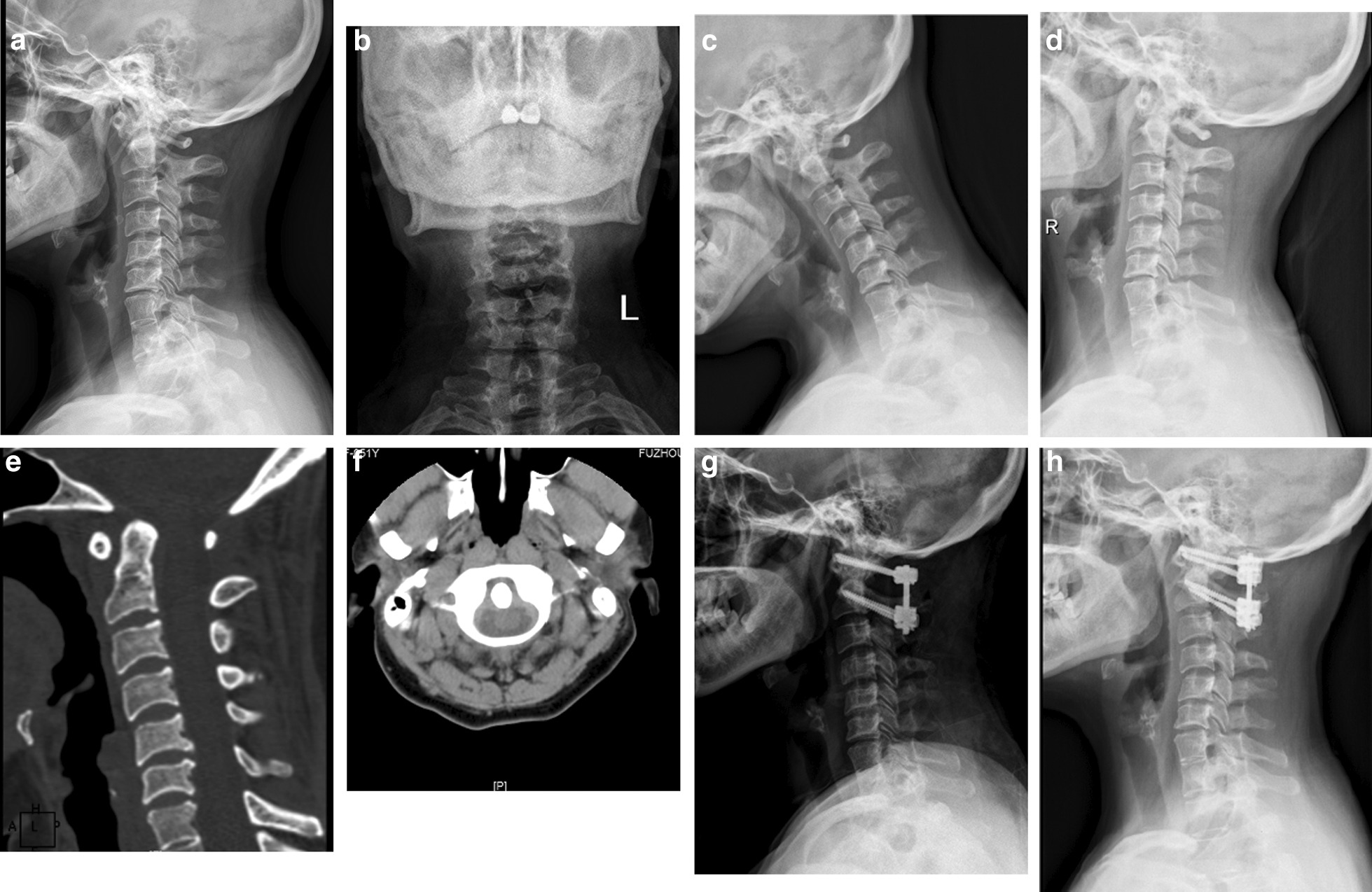
Atlantoaxial dislocation (Type II), female, 51 years old. **a**, **b** Lateral and open-mouth view radiographs of cervical spine showed that the C2–C7 angle was 8°. **c**, **d** Flexion–extension radiographs of cervical spine indicated a partially reducible dislocation of the atlantoaxial joint. **e**, **f** CT scan of cervical spine also indicated the atlantoaxial dislocation. **g** One month after operation, lateral radiograph of cervical spine showed that the dislocation had been fully reduced, and the C2–C7 angle was 8°. **h** Seven months after operation, lateral radiograph of cervical spine showed the loss of subaxial lordosis and the C2–C7 angle was 2°

The cervical sagittal parameters of preoperative and final follow-up between two groups were compared, the preoperative C2–C7 angle of the subaxial lordosis loss group was bigger than the subaxial lordosis increase group (27.6° ± 10.5° vs 10.5° ± 10.5°, *P* < 0.05), but there was no statistical difference in other parameters (Table 2). Univariate chi-square analysis showed that reduction in subaxial lordosis after posterior atlantoaxial fusion was associated with preoperative C2–C7 angle ≥ 20° (*χ*^2^ = 4.923, *P* = 0.026). However, logistic regression analysis showed that the preoperative C2–C7 angle ≥ 20° was not an independent risk factor (OR = 0.147, *P* = 0.225). These results are illustrated in Tables 3, 4. A typical case is shown in Fig. 2.

**Table 2:** Comparison of the cervical sagittal parameters between two groups

	Pre-operation		Final follow-up	
	Loss of subaxial lordosis	Increase in subaxial lordosis	Loss of subaxial lordosis	Increase in subaxial lordosis
C0–C2 (°)	17.4 ± 18.0	23.2 ± 16.3	26.2 ± 10.8	29.1 ± 8.1
C1–C2 (°)	14.2 ± 19.8	11.8 ± 17.6	19.6 ± 8.7	16.7 ± 6.9
C2–C7 (°)	27.6 ± 10.5	10.5 ± 10.5^#^	11.6 ± 12.5	18.3 ± 10.4
C0–C7 (°)	44.8 ± 25.5	33.7 ± 17.2	37.8 ± 14.1	47.6 ± 12.2
C2–C7 SVA (mm)	5.6 ± 12.2	16.5 ± 14.9	16.4 ± 17.0	4.6 ± 9.9
T1S (°)	25.8 ± 7.6	21.7 ± 8.4	22.6 ± 11.5	23.9 ± 5.5
NT (°)	49.4 ± 9.0	51.3 ± 10.0	54.6 ± 9.7	50.9 ± 8.8
TIA (°)	75.2 ± 13.9	73.0 ± 10.4	77.2 ± 10.8	74.7 ± 10.1

^#^Compared with loss of subaxial lordosis, *P* < 0.05

**Table 3:** Univariate analysis of correlation between clinical factors and loss of subaxial lordosis

	Loss of subaxial lordosis (*n* = 5)	Increase in subaxial lordosis (*n* = 13)	*χ*^2^	*P*
*Gender*			0.678	0.410
Male	3	5		
Female	2	8		
*Age (years old)*			0.209	0.648
< 60	4	9		
≥ 60	1	4		
*Preoperative T1S*			0.004	0.952
< 25°	3	8		
≥ 25°	2	5		
*Preoperative C2–C7 SVA*			3.545	0.060
< 15 mm	4	9		
≥ 15 mm	1	4		
*Preoperative TIA*			0.138	0.710
< 70°	2	4		
≥ 70°	3	9		
*Preoperative C2–C7 angle*			4.923	0.026
< 20°	1	10		
≥ 20°	4	3		
*Preoperative C0–C2 angle*			0.780	0.377
< 20°	2	7		
≥ 20°	3	6		
*Preoperative C0–C7 angle*			1.298	0.255
< 45°	2	9		
≥ 45°	3	4		
*Postoperative C1–C2 angle*			2.215	0.137
< 20°	2	10		
≥ 20°	3	3

**Table 4:** Multivariate analysis of correlation between clinical factors and loss of subaxial lordosis

	Multivariate analysis		
	OR	*P*	95% CI of OR
Preoperative C2–C7 SVA	0.147	0.225	0.007–3.264
Preoperative C2–C7 angle	9.138	0.143	0.475–175.879
Postoperative C1–C2 angle	0.012	0.268	0.365–148.158

## Discussion

Cervical sagittal balance plays a decisive role in maintaining the biomechanical properties and normal motor functions of the cervical spine. Although clinical outcomes of posterior atlantoaxial fusion were satisfactory, postoperative cervical sagittal imbalance may be one of the causes of postoperative pain and function loss. More recently, restoring sagittal alignment of cervical spine has received increasing attention, correlating with preoperative surgical planning and postoperative outcomes evaluating.

Parameters used to evaluate the sagittal alignment of the cervical spine include C0–C2 angle, C1–C2 angle, C2–C7 angle, SVA, T1S, NT and TIA [17–19]. There is certain correlation between parameters, the mathematical relationship between these parameters is described by: TIA = T1S + NT. TIA is constant, and theT1S and NT are positional. TIA remains stable with increasing age during adulthood, and does not change with postural, position or spinal degeneration. T1S changes with SVA and kyphotic curvature of the upper thoracic spine. To prevent or limit sagittal imbalance, the cervical spine may also compensate by increasing NT and decreasing T1S in an attempt to maintain horizontal gaze. There were negative correlations between C1–C2 angle and C2–C7 angle as well as between C0–C2 angle and C2–C7 angle in patients with atlantoaxial instability or dislocation. And the negative correlations remain in patients who underwent posterior atlantoaxial fusion [4, 12, 20]. In this study, no significant changes of the cervical sagittal parameters were noted between the last follow-up and before surgery. Therefore, we believe that the posterior atlantoaxial fusion for atlantoaxial dislocation does not affect the cervical sagittal parameters.

The class boundaries of each factors in the univariate analysis of this study were determined mainly by reference to previous research results. Iyer et al. [21] retrospectively analyzed 120 American adult volunteers without cervical and back symptoms, and measured that the average TIA was about 79.8° and T1S was 26.1°. Lee et al. [22] measured an average TIA of 69.5° and T1S of 25.7° in 77 asymptomatic Korean adult volunteers. Based on the measurement results of the above studies, 70° was chosen as the class boundary for preoperative TIA and 25° for preoperative T1S. Lee et al. [22] measured an average C0–C2 of about 22.4° and C2–C7 of 9.9° in 77 asymptomatic Korean adult volunteers. Hardacker et al. [23] studied 100 healthy American adults and found that the C0–C2 angle averaged about 30° and the C2–C7 angle averaged about 9.6°. Harrison et al. measured C2–C7 angle of 17° and 26° by Cobb method and Harrison posterior tangent method on thirty lateral cervical radiographs. Based on these measurement results, the preoperative C2–C7 angle, preoperative C0–C2 angle and preoperative C0–C7 angle were selected as the boundary of 20°, 20° and 45°, respectively. The C2–C7 SVA of normal healthy adults is about 20 mm, but there are differences between people of different ages: 28.5 mm for adults aged 20 to 39 years, 18.2 mm for adults aged 40 to 59 years, − 22.4 mm for adults aged 60 years or older [10, 11]. Regarding the age distribution of our study (average age of 49.6 years), 15 mm was chosen as the class boundary for preoperative C2–C7 SVA. To maintain the physiologic sagittal alignment of the subaxial cervical spine, C1–C2 should be fixed at an optimal fusion angle of 10°-20°. When the fusion angles of C1–C2 were more than 20°, the subaxial lordosis would be insufficient, resulting in degeneration of the lower cervical disk. There was a linear negative association between the C1–C2 fixation angle and the C2–C7 postoperative angle. Therefore, 20° was chosen as the class boundary for the postoperative C1–C2 angle.

Studies have suggested that abnormal changes of lower cervical curvature after atlantoaxial fusion may be related to age, C1–C2 fixation angle and internal fixation techniques. Passias et al. [24] found that age was an important independent factor and negatively correlated with the changes in the curvature of the lower cervical spine after atlantoaxial fusion, which may be due to the fact that the lower cervical spine is more flexible in young people, so that the lower cervical spine can better compensate for the changes in the upper cervical spine, so as to ensure the local balance of the cervical spine. However, the results of this study showed that there was no significant correlation between age and subaxial lordosis loss.

Toyama et al. [5] found that postoperative cervical kyphosis or swan-neck deformity occurred if C1–C2 fixation angle exceeded 30°. Kato et al. [25] retrospectively analyzed the relation between the preoperative C1–C2 angle and C2–C7 angle in 28 consecutive rheumatoid arthritis patients, and found that complete or overreduction in the C1–C2 angle may cause reduced C2–C7 angle and cervical malalignment (lordosis loss, kyphosis, and swan-neck deformity) in patients with a preoperative C1–C2 angle of < 20 degrees. However, the results of this study suggest that postoperative C1–C2 angle didn’t affect subaxial cervical alignment. Atlantoaxial transarticular facet screw fixation (Magerl technique) and C1 lateral mass screws combined with C2 pedicle screws fixation (Harms technique) are the most commonly used techniques for posterior internal fixation in the upper cervical spine. Studies showed that Magerl technique combined with posterior titanium atlantoaxial cable fixation was more likely to cause excessive lordosis position of C1–C2 than Harms technique, resulting in postoperative kyphosis deformity of the lower cervical spine. Therefore, we chose Harms technique to reduce the occurrence of postoperative kyphosis deformity. In this study, the univariate Chi-square analysis showed that postoperative subaxial lordosis was negatively correlated with preoperative C2–C7 angle, that is, preoperative C2–C7 angle ≥ 20° could easily cause postoperative loss of subaxial lordosis. A large preoperative C2–C7 angle would lead to a small preoperative C1–C2 angle in an attempt to maintain horizontal gaze. There were negative correlations between C1–C2 angle and C2–C7 angle [4, 12, 20], thus partial reduction in the C1–C2 angle after posterior atlantoaxial fusion (increased from 12.4° preoperatively to 17.5° postoperatively) may cause reduce of C2–C7 angle. However, the multivariate logistic regression analysis in this study showed that the preoperative C2–C7 Angle ≥ 20° was not an independent risk factor (OR = 0.147, *P* = 0.225), indicating that there may be other factors affecting the postoperative loss of subaxial lordosis. Limitations of the study include the small sample size, lack of long-term follow-up. Furthermore, whether postoperative pain or muscle stiffness has an impact on the results needs to be further clarified.

In conclusion, our study demonstrated that subaxial lordosis loss may occur after posterior atlantoaxial fusion, and preoperative C2–C7 angle ≥ 20° was a risk factor of postoperative loss of subaxial lordosis. To avoid postoperative cervical malalignment for patients with atlantoaxial dislocation, more attention should be paid to fully consider the age, etiology and other factors before surgery, determine the appropriate atlantoaxial fixation angle and surgical technique.

## Data Availability

All data are available on request.
